# Supplementary material for: Associations between Normal Organs and Tumor Burden in Patients Imaged with Fibroblast Activation Protein Inhibitor-Directed Positron Emission Tomography
Source: Cancers (Basel). 2022 May 25;14(11):2609. doi: 10.3390/cancers14112609 (PMC9179441; doi:10.3390/cancers14112609)
Supplement: Supplementary file 1 [file cancers-14-02609-s001.zip › cancers-1730940-supplementary.pdf]

Table S1: Descriptive statistics of uptake in surgical routes or post-surgical scarring.

	Parameter	Minimum	Median	Maximum	Mean[1]	SD[1]
**Post-surgical uptake**	SUV_{max}	5.19	9.92	16.0	9.80	3.29
	PS-FV	6.80	37.0	111	47.3	31.2
	PS-FFA	23.4	168	767		

[1] mean and standard deviation (SD) are only shown for normally distributed data. SUV_{max} = maximum standardized uptake value, PS-FV = post-surgical FAP uptake volume, PS-FFA = post-surgical fractional FAP activity, defined as mean standardized uptake value × PS-FV).

Table S2. Correlation (Spearman's Rho, ϱ; or Pearson's r) to determine associations between radiotracer uptake in normal organs and post-surgical scarring.

			Post-surgical scarring		
			**SUV_{max}**	**PS-FV**	**PS-FFA**
**Normal Organs**	Heart	ϱ(/r*)	0.49*	-0.30*	0.23
		P	0.06	0.28	0.40
	Bone Marrow	ϱ(/r*)	0.02*	0.35*	0.12
		P	0.94	0.20	0.66
	Liver	ϱ	0.00	0.06	0.03
		P	>0.99	0.84	0.92
	Spleen	ϱ	-0.46	-0.30	-0.48
		P	0.10	0.29	0.09
	Pancreas	ϱ	-0.27	0.01	-0.24
		P	0.34	0.98	0.40
	Right Kidney	ϱ	0.23	-0.18	-0.04
		P	0.40	0.53	0.89
	Left Kidney	ϱ	0.29	0.03	0.24
		P	0.29	0.91	0.40

* for normally distributed data, Pearson's r is shown. SUV_{max} = maximum standardized uptake value, PS-FV = post-surgical FAP uptake volume, PS-FFA = post-surgical fractional FAP activity, defined as mean standardized uptake value × PS-FV).

Table S3 Descriptive statistics of uptake in normal organs and tumor lesions ('high-tumor burden' only, n = 25).

		Parameter	Minimum	Median	Maximum	Mean[1]	SD[1]
**Normal Organs**	Heart	SUV_{mean}	0.48	1.11	2.12	1.16	0.43
	BM	SUV_{mean}	0.35	0.54	0.94	0.58	0.15
	Liver	SUV_{mean}	0.31	0.70	1.40	0.75	0.29
	Spleen	SUV_{mean}	0.55	0.86	1.26	0.86	0.20
	Pancreas	SUV_{mean}	1.05	2.11	7.39		
	Kidney R	SUV_{mean}	0.86	1.37	2.47	1.50	0.39
	Kidney L	SUV_{mean}	0.86	1.48	2.60	1.57	0.47
**Tumor Burden**		SUV_{max}	6.77	10.7	34.4		
		TV	17.4	129.3	838		
		FTA	84.3	603.9	7155		

[1] mean and standard deviation (SD) are only shown for normally distributed data. BM = bone marrow, R = right, L = left, SUV_{mean} = mean standardized uptake value, SUV_{max} = maximum standardized uptake value, TV = tumor volume, FTA = fractional tumor activity, defined as mean standardized uptake value × TV).

Table S4 Correlation (Spearman's Rho, ϱ) to determine associations between radiotracer uptake in normal organs and tumor lesions ('high tumor burden' only, n = 25).

			'High' Tumor Burden		
			**SUV_{max}**	**TV**	**FTA**
**Normal Organs**	Heart	ϱ	0.44	-0.34	-0.11
		P	0.03	0.09	0.60
	Bone Marrow	ϱ	0.27	0.16	0.22
		P	0.21	0.48	0.32
	Liver	ϱ	0.31	0.31	0.47
		P	0.13	0.13	0.02
	Spleen	ϱ	-0.03	0.19	0.10
		P	0.87	0.35	0.64
	Pancreas	ϱ	0.23	0.16	0.27
		P	0.30	0.47	0.23
	Right Kidney	ϱ	0.09	0.13	0.23
		P	0.67	0.53	0.22
	Left Kidney	ϱ	-0.06	0.11	0.12
		P	0.78	0.60	0.57

SUV_{max} = maximum standardized uptake value, TV = tumor volume. FTA = fractional tumor activity.

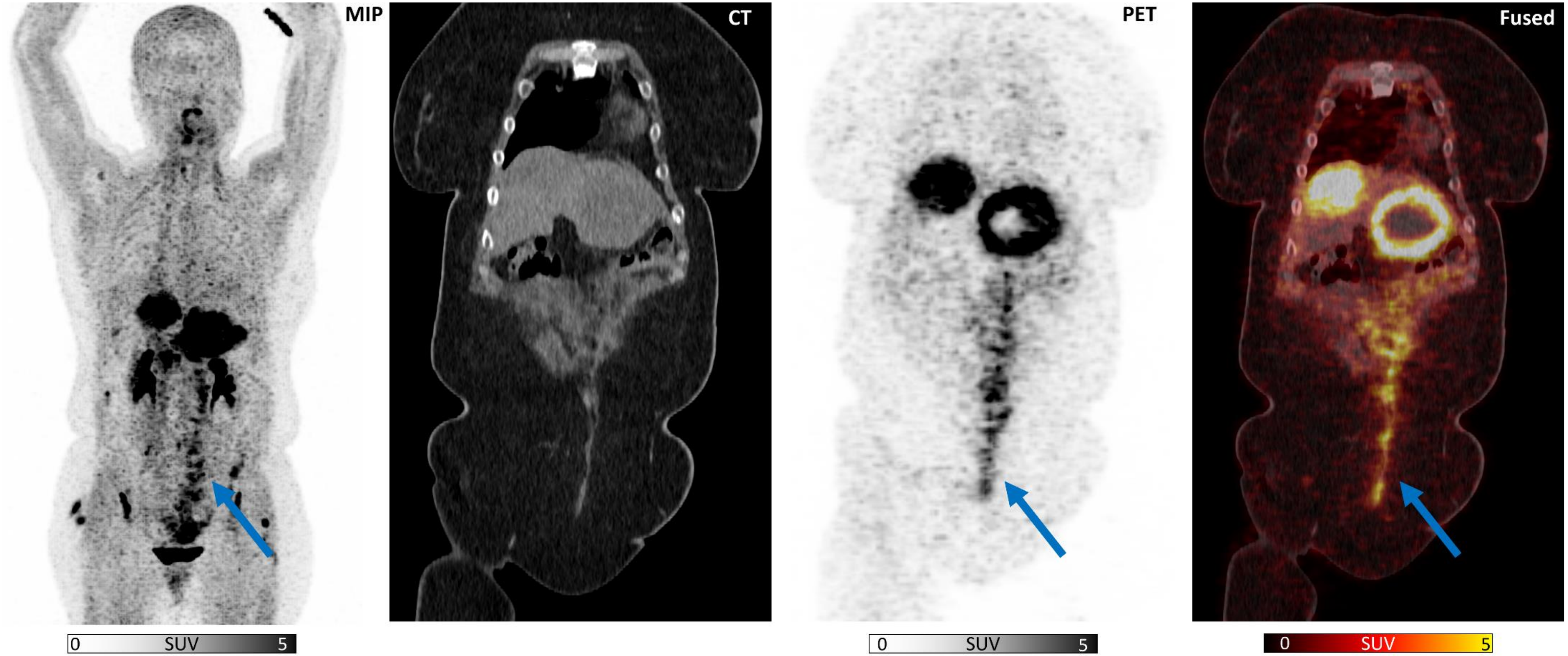

Figure S1. [^{68}Ga]Ga-FAPI-PET in a patient with post-surgical scarring after median laparotomy. Blue arrows indicate increased tracer uptake in the surgical route.
